# A new genus and species of Metopiinae (Hymenoptera, Ichneumonidae) from Mexico

**DOI:** 10.3897/zookeys.207.3339

**Published:** 2012-07-11

**Authors:** Andrey I. Khalaim, Enrique Ruíz-Cancino, Juana M. Coronado-Blanco

**Affiliations:** 1 División de Estudios de Postgrado e Investigación, Facultad de Ingeniería y Ciencias, Universidad Autónoma de Tamaulipas, Cd. Victoria 87149, México; 2 Zoological Institute, Russian Academy of Sciences, Universitetskaya nab. 1, St. Petersburg 199034, Russia

**Keywords:** North America, *Ojuelos*, new genus, new species, taxonomy

## Abstract

A new genus of Metopiinae, *Ojuelos* Khalaim, **gen. n.**, with a single species, *Ojuelos juachicus* Khalaim & Ruíz-Cancino, **sp. n.**, is described from Jalisco Province in central Mexico. *Ojuelos* belongs to the group of three genera (*Cubus* Townes & Townes, *Colpotrochia* Holmgren and *Triclistus* Förster) having a high lamella separating the antennal sockets and reaching the median ocellus (this lamella possesses a dorsal groove in it, just below the median ocellus), but differs from these genera primarily by 1) face and clypeus not convex in lateral view, 2) face separated from clypeus by a shallow transverse impression, 3) mandible with lower tooth very strongly reduced, 4) flagellomeres of antenna strongly transverse, and 5) dorsal carinae of propodeum reduced. A portion of the key to the genera of Metopiinae is provided to distinguish the new genus.

## Introduction

Metopiinae is a moderately large subfamily with over 750 described extant species distributed in 25 genera (Yu et al. 2005). The subfamily is well represented in all parts of the World, occurs from the Arctic to the tropics, and some large genera (e.g. *Exochus* Gravenhorst, *Metopius* Panzer) are almost worldwide. Species of Metopiinae can be collected in various habitats but generally are most abundant in forests. All known Metopiinae are solitary endoparasitoits of Lepidoptera. Oviposition is into the exposed or concealed host larva, and emergence is always from the pupa (Townes 1971; Gauld and Sithole 2002).

Most Metopiinae species are easily recognized by their face and clypeus which are confluent and not separated by a groove and moderately to strongly convex in lateral view (except that the face of *Metopius* is flat and with a large shield-shaped area bounded by a sharp carina). Most have the upper margin of the face produced dorsally between the antennal insertions into a triangular flange. The body and legs are usually robust, the metasoma is more or less cylindrical, and the ovipositor is thin and very short (not projecting beyond the apex of metasoma). Some Orthocentrinae [Orthocentrus group of genera sensu Wahl and Gauld (1998)] have a similar structure of the face and clypeus (confluent and strongly convex) but these may be distinguished from Metopiinae primarily by the long cylindrical scape of antenna and because the upper edge of the face lacks the triangular interantennal projection. In addition, Orthocentrinae parasitize Mycetophiloidea (Diptera) (Townes 1971).

A list of Mexican Metopiinae was prepared by Ruíz-Cancino et al. (2002) and included only 17 species from 7 genera. Some more metopiine species and genera were mentioned from Mexico in the book on Costa Rican Ichneumonidae published in the same year by Gauld and Sithole (2002). Five new records of metopiine species were recently recorded from southern Mexico by González-Moreno et al. (2011), and a review of the Mexican species of *Colpotrochia* Holmgren (five species of this genus are recorded for the first time) and *Cubus* Townes & Townes was published by Khalaim and Ruíz-Cancino (2011). Nevertheless at present, the Mexican Metopiinae are very poorly studied, and only a small part of the real Mexican metopiine fauna is described. For comparison, the fauna of America north of Mexico comprises about 145 species from 19 genera (Townes and Townes 1959), and 130 species from 14 genera were recorded from Costa Rica (Gauld and Sithole 2002). Here a new metopiine genus with a new species is described from central Mexico.

## Material and methods

Wing venation and morphological terms predominantly follow Gauld and Sithole (2002). Photographs were taken at the Zoological Institute of the Russian Academy of Science (St. Petersburg, Russia) with a DFC 290 digital camera attached to a Leica MZ16 stereomicroscope, and images were combined using Helicon Focus software. Generic identification was checked through the keys to world genera of Metopiinae (Townes 1971), genera of America north of Mexico (Townes and Townes 1959), genera of Costa Rica (Gauld and Sithole 2002) and genera of Australia (Fitton 1984). The only specimen of the new genus was collected on herbs in a dry area above 2200 m in central Mexico. The holotype of *Ojuelos juachicus* is deposited at the Insect Museum of Universidad Autónoma de Tamaulipas, Cd. Victoria, Mexico (UAT).

## Results

### Key to world genera of Metopiinae (modified from Gauld and Sithole 2002, Townes 1971)

**Table:** 

1	Face with a large shield-shaped area bounded by a sharp carina. Tergites 1 and 2 fused (clearly visible in lateral view). Laterotergite 1 broad	*Metopius* Panzer
–	Face more or less convex, without a concave shield-shaped area. Tergites 1 and 2 separated by a flexible membrane	2
2	Interantennal process of face produced dorsally into a high longitudinal lamella separating the antennal sockets; this lamella with a longitudinal groove in the dorsal surface, just below the median ocellus (Fig. 7)	3
–	Interantennal process of face not produced dorsally as a longitudinal lamella, or if a weak, short lamella present, it does not have groove in the dorsal surface	Other genera of Metopiinae
3	Propleuron almost cubical in profile, resembling fore coxa in shape. Mesosternal region with a pair of flattened finger-like processes projecting backwards over bases of mid coxae. Upper half of mesopleuron strongly inflated	*Cubus* Townes & Townes
–	Propleuron not cubical in profile. Mesosternal region smooth or with a weak lamella before mid coxae, without finger-like processes. Mesopleuron laterally usually not strongly inflated	4
4	Face and clypeus not especially convex in lateral view, separated by weak and broad transverse impression. Mandible with lower tooth strongly reduced, represented as a small tubercle; upper tooth large, chisel-shaped (Fig. 5). Propodeum with only area posteroexterna bounded by carinae, otherwise ecarinate (Fig. 10). Flagellomeres of antenna very short, mid flagellomeres almost twice as broad as long (Fig. 2)	*Ojuelos* Khalaim, gen. n.
–	Face and clypeus moderately to very strongly convex in lateral view; clypeus not separated from face. Mandible with lower tooth shorter, subequal to or longer than upper tooth, not especially reduced, and upper tooth more or less pointed, not chisel-shaped. Propodeum with carinae not as above. Flagellomeres of antenna more or less elongate	5
5	First metasomal segment weakly to quite strongly tapered anteriorly, but always evenly so, thus the anterior 0.4 is not parallel sided; its spiracle near its anterior 0.3. Sternite 1 short, at most reaching about 0.3 of length of tergite. Mandible with lower tooth much shorter than upper tooth	*Triclistus* Foerster
–	First metasomal segment petiolate anteriorly, the anterior 0.4 slender and parallel sided; its spiracle near, at or behind the centre. Sternite 1 long, reaching more than 0.5 of length of tergite. Mandible with teeth subequal or with lower tooth longer than upper tooth	*Colpotrochia* Holmgren

#### Ojuelos

Khalaim gen. n.

urn:lsid:zoobank.org:act:6A8BDDD3-BF85-458A-A5F4-9850741A1819

http://species-id.net/wiki/Ojuelos

Figs 1 [Fig F2] 13

##### Type species:

*Ojuelos juachicus* Khalaim & Ruíz-Cancino, sp. n.

##### Composition.

The new genus contains only the type species, *Ojuelos juachicus*, described below.

##### Diagnosis.

The new genus belongs to the group of three genera (*Cubus*, *Colpotrochia* and *Triclistus*) having a characteristic lamella separating the antennal sockets and reaching the median ocellus (this lamella possesses a longitudinal groove in it, just below the median ocellus) (Fig. 7), and can be distinguished from these genera by the characters given in the key above. Besides characters mentioned in the key, *Ojuelos* differs from *Colpotrochia* by the first metasomal segment not petiolate and evenly tapered anteriorly (Fig. 11), and lower tooth of mandible very small (Fig. 5), and from *Triclistus* by the subgenital plate of female without a deep V-shaped invagination posteriorly (Fig. 13) (some species of *Triclistus* also have hypopygium only weakly emarginate).

*Ojuelos* can be distinguished from all other genera of Metopiinae by combination of the following characters: 1) frons with a medial, longitudinal lamella that possesses a groove in dorsal surface (Fig. 3); 2) face very weakly convex in profile and separated from clypeus by a weak groove; 3) first metasomal tergite in dorsal view gradually widening posteriorly (Fig. 11) (not petiolate as in *Colpotrochia*); 4) mesosternum without a pair of flattened, finger-like processes projecting posteriorly over bases of mid coxa (projections present in *Cubus*); 5) propodeum lacks most carinae, only the area posteroexterna is bounded by carinae, whereas the longitudinal carinae are absent anteriorly.

##### Description.

Fore wing length 9.5 mm, body length about 12.6 mm. Body and legs predominantly black, flagellum yellowish brown, wings yellow with distal margin broadly infuscate (Fig. 8).

**Head:** Mandibles stout, not twisted, with lower tooth strongly reduced (very small and inconspicuous) and upper tooth broad and chisel-shaped (Fig. 5). Labrum exposed, short and apically truncated (Fig. 6). Malar space half as long as basal width of mandible. Clypeus 3.0 times as broad as high, more or less flat in lateral view, separated from face by weak and broad transverse impression (Fig. 5). Face and upper 0.8 of clypeus very densely and coarsely punctate (punctures partly merging). Face very weakly convex, with upper part produced dorsomedially into a triangular projection that extends posteriorly as a thin, longitudinal lamella between bases of antennae; this lamella reaching to the median ocellus, and dorsally with a conspicuous groove (Fig. 7). Ocelli not enlarged. Back of head steeply declivous behind the posterior ocelli. Occipital carina dorsally close to foramen magnum, almost complete but obliterated ventrally before hypostomal carina (Fig. 4). Hypostomal carina strong, raised into a high flange (Fig. 4). Flagellum of antenna rather short and thick; all flagellomeres, excepting three basal and one apical flagellomere, distinctly transverse (Fig. 2).

**Mesosoma:** Propleuron not enlarged. Epomia very sharp and strongly raised, close to anterior margin and extending upwards (Fig. 7). Notauli completely absent. Mesopleuron strongly inflated (lateral sides of mesopleuron conspicuously protuberant in dorsal view). Epicnemial carina reaching almost margin of pleuron immediately below the subalar prominence, with a secondary carina extending from pleural margin near lower corner of pronotum, to join the subtegular ridge. Sternaulus absent. Posterior transverse carina of mesopleuron present laterally and absent ventrally. Propodeum rather short, convex, almost ecarinate, with only area posteroexterna clearly bounded by carinae, and with weak and short longitudinal carina extending anteriorly from anterior margin of area posteroexterna (Fig. 10). Propodeal spiracle large and oval (Fig. 10). Pleural carina between propodeum and metapleuron complete. Submetapleural carina complete, strongly raised anteriorly.

**Wings:** Fore wing with stalked rhombic areolet (Fig. 8), vein *cu-a* strongly inclivous and distad *Rs*&*M*. Vein 2*m-cu* slightly S-curved, with one long bulla. Hind wing with distal abscissa of *Cu*1 distinct, meeting cu-a much closer to 1*A* than to *M*.

**Legs:** Robust, all femora thickened. Fore tibia without apical tooth. Hind and mid tibiae with two spurs; inner spur of hind tibia longer than outer spur (Fig. 12). Apical tarsomeres not swollen. Tarsal claws large, simple but with long hairs.

**Metasoma:** Tergites 1 and 2 of metasoma separated. First tergite 1.6 times as long as posteriorly broad (length measured from hind margin of propodeum), evenly tapered anteriorly in dorsal view (Fig. 11), with spiracle near its anterior 0.35 (Fig. 9); dorsomedian carinae virtually absent, dorsolateral carinae distinct only at base of the tergite, completely absent behind spiracle; sternite 1 reaching about 0.33 of length of tergite. Tergites 2 and 3 with neither dorsal nor dorsolateral carinae. Laterotergites 2 and 3 narrow, separated by a sharp crease. Laterotergite 4 separated from tergite by weak crease only anteriorly. Subgenital plate of female roundly truncated and very weakly concave at apex (Fig. 13). Ovipositor thin and short, slightly upcurved, without dorsal subapical notch, slightly projecting beyond apex of subgenital plate (Fig. 12).

##### Etymology.

Named after the type locality, Ojuelos de Jalisco. Gender masculine.

#### Ojuelos juachicus

Khalaim & Ruíz-Cancino sp. n.

urn:lsid:zoobank.org:act:2F287E7F-CF27-4383-BDF4-3DF74EA9EB43

http://species-id.net/wiki/Ojuelos_juachicus

Figs 1 [Fig F2] 13

##### Description.

Female.

**Head:** Mandibles mostly densely and coarsely punctate, very finely shagreened at apex. Labrum 6.3 times as long as basally broad (Fig. 6). Maxillary and labial palpi slender and moderately long. Clypeus with lower margin truncated (Fig. 6), smooth in lower 0.2. Face with weak median tubercle in its upper part. Inner eye orbits moderately concave at level of antennal insertions. Vertex and genae with dense and sharp punctures (distance between punctures mostly equal to or slightly longer than diameter of puncture), smooth between punctures; punctures in lower part of genae sparser. Gena, in dorsal view, rounded, about as long as eye width (Fig. 3). Lateral ocellus separated from eye margin by distance equal to maximum diameter of ocellus. Flagellum of antenna with 46 flagellomeres; the basal flagellomere about 2.5 times as long as broad; flagellomeres 4+ distinctly transverse, mid flagellomeres almost twice as broad as long (Fig. 2).

**Mesosoma:** Pronotum mediodorsally smooth, laterally deeply concave. Mesoscutum weakly convex, posterolaterally with a sharp flange, entirely densely and sharply punctate, smooth between punctures. Scutoscutellar groove deep and smooth. Scutellum moderately convex in lateral view, smooth, with sharp punctures and with lateral carinae only at its extreme base. Mesopleuron mostly densely and sharply punctate, smooth and impunctate only posteriorly. Propodeum dorsally polished, laterally and posteriorly finely punctate to coriaceous. Metapleuron smooth, in dorsoposterior 2/3 with sparp punctures, in ventroanterior 1/3 impunctate.

**Metasoma:** Tergite 2, in dorsal view, slightly transverse; laterotergite 2 parallel-sided, 3.8 times as long as broad. Tergite 3 with laterotergite rather narrow and parallel-sided. Tergite 1 dorsally predominantly finely and densely punctate, smooth and shining between punctures. Tergites 2–6 dorsally mostly finely and very densely punctate, weakly polished between punctures. Subgenital plate finely punctate.

**Figure 1.**
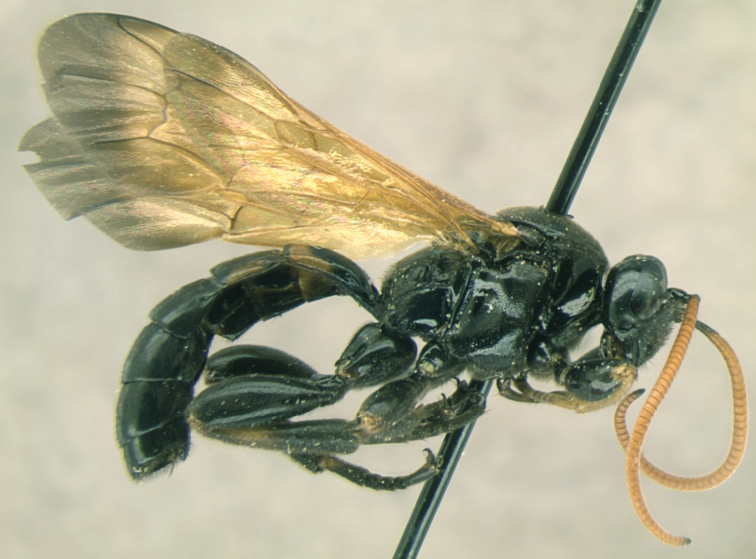
*Ojuelos juachicus* Khalaim & Ruíz-Cancino, **sp. n.**, habitus, female holotype.

**Figures 2–8. F2:**
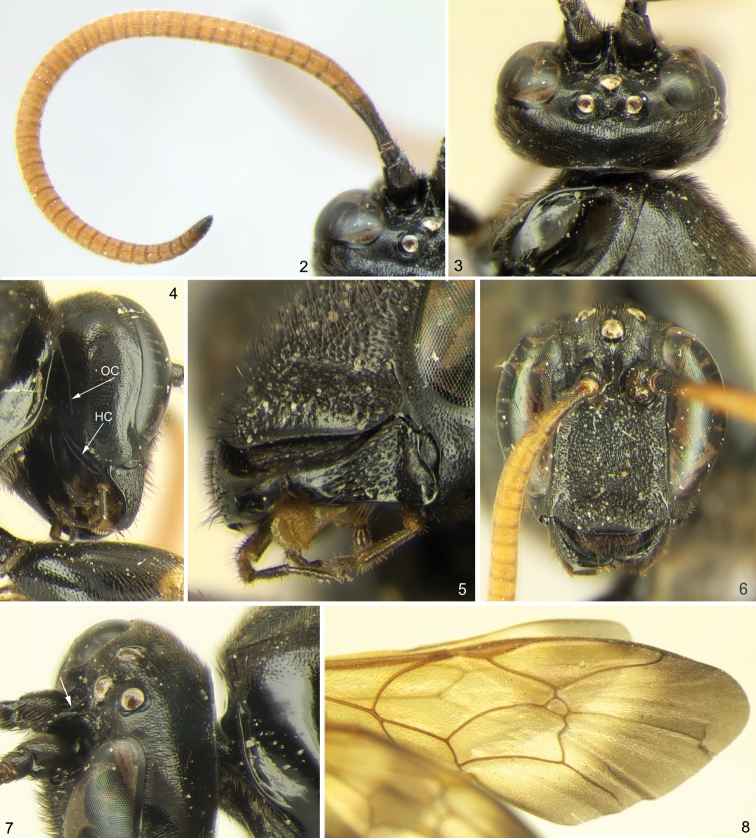
*Ojuelos juachicus* Khalaim & Ruíz-Cancino, **sp. n.** **1** antenna, dorsal view **2** head, dorsal view **3** head, latero-ventro-posterior view (OC – occipital carina, HC – hypostomal carina) **5** head, latero-ventro-anterior view **6** head, frontal view **7** head and anterior part of mesosoma, dorsolateral view **8** fore wing, dorsal view.

**Coloration:** Body and legs almost entirely black (Fig. 1). Flagellum yellowish brown with the basal and the apical flagellomeres fuscous (Fig. 2). Mouth parts blackish. Wings yellow with distal margin broadly infuscate, pterostigma and most of veins pale brown (Fig. 8). Legs predominantly black; fore leg with femur yellowish brown apically, tarsus yellowish brown, partly infuscate; mid leg with femur apically, tibia basally and basitarsus basally yellowish brown; hind leg with tibia with broad subbasal yellowish brown band and basitarsus basally yellowish brown. Tergite 1 with broad posterior brownish yellow band; tergite 2 laterally yellowish brown (Fig. 11).

Male unknown.

**Material examined.** Holotype female, México, Zacatecas Prov., 20 km S Ojuelos de Jalisco, Juachí, La Papa de Arriba, 21°42.104'N, 101°36.791'W, 2275 m, sweeping, 21.IX.2011, coll. A.I. Khalaim (UAT).

**Distribution.** Central Mexico (Jalisco).

**Etymology.** Named after the type locality, Juachí.

**Figures 9–13.**
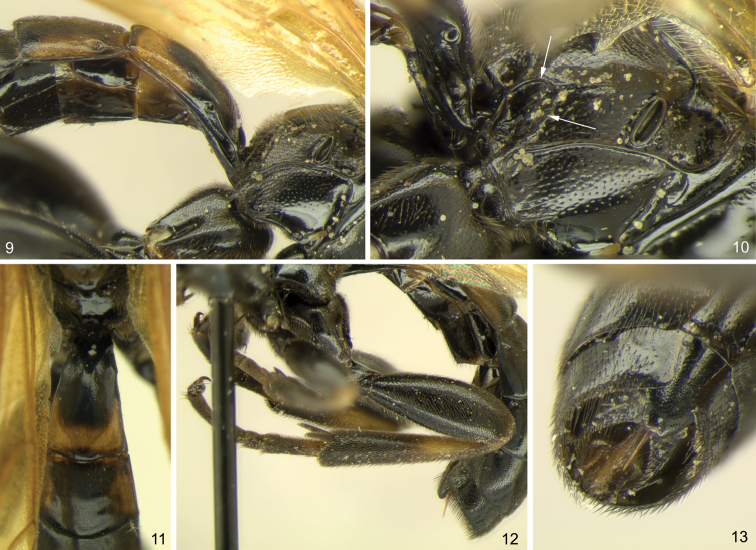
*Ojuelos juachicus* Khalaim & Ruíz-Cancino, **sp. n.** **9** propodeum, metapleuron and metasomal segments 1–2, lateral view **10** propodeum and metapleuron, lateral view **11** propodeum and metasomal segments 1–2, dorsal view **12** metasoma and hind leg, lateral view **13** apex of metasoma, latero-ventro-posterior view.

## Discussion

*Ojuelos* is a distinct member of the Metopiinae as it has a characteristic stout body and legs, cylindrical metasoma with short first segment, face weakly separated from clypeus and with the dorsally projecting triangular interantennal flange, lack of sternaulus and tooth on apex of fore tibia, and having a short and very slender ovipositor.

We place *Ojuelos* to the group of three genera, *Cubus*, *Colpotrochia* and *Triclistus* (all occur in Mexico), based on the putative synapomorphy of the interantennal lamella that bears a dorsal, longitudinal groove. The genus is distinguished from other genera of the subfamily primarily by the rather unusual structure of the head which has the face and clypeus only slightly convex in lateral view and separated by a weak impression. Within the subfamily similar head present only in two small genera, the predominantly Holarctic *Periope* Haliday (Kusigemati 1968) and the Holarctic and Neotropical *Scolomus* Townes & Townes (= *Apolophys* Townes) (Gauld and Wahl 2006). *Ojuelos* differs from *Periope* by having a hind tibia with two robust spurs (one slender spur in *Periope*) and a strongly reduced lower tooth of the mandible, from *Scolomus* by having the triangular interantennal projection of face (in *Scolomus* face without triangular projection dorsally), a shorter malar space (1.2–1.8 times as long as basal width of mandible in *Scolomus*), a strongly transverse clypeus (subquadrate, about as broad as long in *Scolomus*), a fore wing with vein *cu-a* strongly distad *Rs*&*M* (opposite to slightly distad in *Scolomus*), and from both genera by having a reduced longitudinal carinae of the propodeum (Figs 10, 11) (propodeum with complete medial longitudinal carinae in *Periope* and more or less developed basal and apical transverse carinae in *Scolomus*).

## Supplementary Material

XML Treatment for Ojuelos

XML Treatment for Ojuelos juachicus
